# Inhibition Effect of a Custom Peptide on Lung Tumors

**DOI:** 10.1371/journal.pone.0109174

**Published:** 2014-10-13

**Authors:** Chih-Yu Huang, Hsuan-Yu Huang, Michael D. Forrest, Yun-Ru Pan, Wei-Jen Wu, Hueih-Min Chen

**Affiliations:** 1 Nano Biomedical Group, National Nano Device Laboratories, National Applied Research Laboratories, Hsinchu, Taiwan; 2 Department of Computer Science, University of Warwick, Coventry, United Kingdom; University of North Carolina School of Medicine, United States of America

## Abstract

Cecropin B is a natural antimicrobial peptide and CB1a is a custom, engineered modification of it. *In vitro*, CB1a can kill lung cancer cells at concentrations that do not kill normal lung cells. Furthermore, *in* *vitro,* CB1a can disrupt cancer cells from adhering together to form tumor-like spheroids. Mice were xenografted with human lung cancer cells; CB1a could significantly inhibit the growth of tumors in this *in* *vivo* model. Docetaxel is a drug in present clinical use against lung cancers; it can have serious side effects because its toxicity is not sufficiently limited to cancer cells. In our studies in mice: CB1a is more toxic to cancer cells than docetaxel, but dramatically less toxic to healthy cells.

## Introduction

Lung cancer is often fatal. Globally, it is the leading cancer in terms of incidence and mortality. In 2012, there were 1.82 million new cases and 1.56 million deaths due to lung cancer [1], [2]. The causes of lung cancer are incompletely understood. However, it has been associated with a number of environmental factors such as cigarette smoke [3], air pollution [4] and contact with certain chemicals (e.g. benzene, dioxins, etc) [5]. Lung cancer has an incredibly high mortality rate; it is often diagnosed too late because it is difficult to detect in its early stages, when it is more curable [6]–[8]. Typically lung cancer patients are diagnosed at either the primary tumor stage or advanced-stage metastases [9], [10]. One way of reducing deaths from lung cancer is to reduce people’s exposure to the aforementioned environmental risk factors. Furthermore, lung cancer can have a genetic component; if someone has a relative that has had lung cancer they may be more predisposed to developing this condition and should be closely monitored. But ultimately, there is an urgent need for a drug that can kill lung cancer cells, and/or halt their proliferation, but that has a low toxicity to non-cancerous cells.

In humans, lung cancer can be divided into two major histopathological groups: non-small-cell lung cancer (NSCLC) [11], [12] and small-cell lung cancer (SCLC) [13], [14]. Approximately 80% of human lung cancers are NSCLC; these cancers can be subdivided into adenocarcinoma, squamous cell carcinoma and large-cell carcinoma [15]–[17]. The 5-year overall survival rates for NSCLC and SCLC are about 14% [18] and 5% [19] respectively.

Treatment options for lung cancer include chemotherapy [20], [21], surgery [21] and radiotherapy [22]. The choice of therapy(s) depends on the stage and status of the disease within the patient. Surgery is used to remove obvious tumors. Chemotherapy is the use of chemicals to kill cancer cells [23]–[26] and it can typically act even if the cancer has spread around the body. However, present chemotherapies produce severe side effects as they aren’t specific enough: they are highly toxic to non-cancerous cells also. Typically, chemotherapy is used in combination with surgery and radiotherapy. Advantageously, this can reduce the amount of exposure a patient needs to chemotherapy [27]. However, NSCLC (80% of lung cancers) have a very limited response rate to current chemotherapeutic agents with a 2-year survival rate of between 10% and 16% [28]. In this paper, we examine an alternative. The use of a customized biological peptide (CB1a) as a prospective therapy for lung cancer.

Peptides are generally small proteins of 50 amino acids or less. In nature there are many cationic, lytic peptides. A variety of organisms produce them as bacteriocins, to protect against invading bacteria. Some of these have been found to be effective against tumor cells *in* *vitro* [29]–[31] and *in* *vivo* [32]. In many cases, such peptides are relatively harmless to normal human cells; including erythrocytes [33]. Cecropin is a cationic, lytic peptide found in silkworms (*Hyalophola cecropia*) [34]. It has broad spectrum inhibitory effect against many human and plant pathogens [29], [35]–[38]. Among the cecropin family (cecropin: A, B, C, D, E and F), cecropin B (CB) is known to have the highest level of antimicrobial activity [34]. Previous studies have shown that CB can lyse not only bacteria, but also cancer cells [29], [39]–[42]. Although, its cancer cell selectivity, and killing efficacy, is not suitable for drug production [33]. However, a custom peptide, CB1a, has been derived from CB and this has much better selectivity and efficacy. CB1a has three repeats of the terminal ten amino acids found at the N-terminus of CB, which are: Lys-Trp-Lys-Val-Phe-Lys-Lys-Ile- Glu-Lys; or KWKVFKKIEK. The second and third repeats are linked by a hinge bridge motif (Ala-Gly-Pro or AGP). A heparin binding motif is the sequence: XBBXBX, where B represents any basic amino acid and X represents any other amino acid [43], [44]. CB1a has a heparin binding motif from its component sequence: EKKWKV.

Previous *in* *vitro* studies have shown that CB1a has a promising activity against several cancer cell lines, including lung cancer cells, but with a low toxicity to normal human cells [33], [45]. This paper confirms these results. *In vitro*, we show that CB1a can kill lung cancer cells at a concentration that does not kill normal lung cells. Secondly, we show that CB1a disrupts adhesions among cancer cells and stops them from aggregating into multi-cellular tumor spheroids (MCTS) *in* *vitro*. We go on in this paper to show that these promising *in* *vitro* results translate to a promising *in* *vivo* action.

Xenotransplantation is the transplantation of cells, tissues or organs - referred to as a xenograft - from one species to another. Nude strain mice have a disrupted FOXN1 gene and this produces a deteriorated or absent thymus, many less T cells/lymphocytes and a compromised immune system. They cannot mount any rejection response to a xenograft. Nude strain mice were subcutaneously transplanted with human lung cancer cells (NCI-H460) at their abdominal flank (a xenograft model). If CB1a was given to the mice for a week before the xeno-transplantation of cancer cells (Pre-treatment) it could prevent tumor growth. If CB1a was given after the xeno-transplantation (Post-treatment) it could inhibit tumor growth. The subcutaneous injection point for CB1a was in the dorsolateral neck area and the xenograft cancer cells were subcutaneously transplanted to the abdominal flank area of the mouse. The distance between these two points is far (∼4 cm) as compared to the length of the mouse (∼6 cm). This distance shows that CB1a can survive in the blood stream long enough to travel to a remote site and exert its anti-cancer action. Further to this, we show that CB1a has a sufficiently long half-life in rat blood.

Drugs presently in use to combat lung cancers have severe side effects because their toxicity is not sufficiently selective to cancer cells. For example, docetaxel administered at a dose of 100 mg/m^2^ in a three-week cycle causes haematological toxicity (86% patients having grade 3 or 4 neutropenia) [21]. Our study in mice suggests that CB1a is much less toxic to normal cells than docetaxel, whilst having a greater toxicity to lung cancer cells.

Xenograft tumor growth in mice, with human NCI-H460 cancer cells, is not completely analogous to human circumstances. However, it has been shown that if a drug can combat such a tumor, it is likely to be successful in human patients [46].

## Materials and Methods

### Ethics Statement

All animal experiments were conducted in a specific pathogen free environment as dictated by the Association for Assessment and Accreditation of Laboratory Animal Care International Guidelines. Four-week old male nude mice (NU/NU) were used (sourced from BioLASCO Taiwan Co., Ltd., Taipei, Taiwan). All experimental protocols were approved by the Animal Care and Utilization Committee, National Nano Device Laboratories, Taiwan, R.O.C.

### Preparation of CB1a peptide

Preparations of natural peptide (CB) and custom peptide (CB1a) have been previously described in detail [31], [33], [47]. The sequences of CB and CB1a are shown below:

CB: NH_2_-KWKVFKKIEK-MGRNIRNGIVK-AGP-AIAVLGEAKAL-COOH.

CB1a: NH_2_-KWKVFKKIEK-KWKVFKKIEK-AGP-KWKVFKKIEK-COOH.

KWKVFKKIEK is an amphipathic α-helix (one side is hydrophilic, other side is hydrophobic); AGP is a hinge bridge. CB has one KWKVFKKIEK sequence, one AGP sequence and 2 other constituent sequences. CB1a has 3 repeats of KWKVFKKIEK and an AGP sequence. CB1a peptide was synthesized by an Applied Biosystems (ABI) peptide synthesizer and purified using reverse-phase high performance liquid chromatography. The purity was about 96%. The molecular weight of generated peptides was investigated by mass spectra and their recorded weight was nearly identical to the theoretical, calculated weight of the desired sequence (4190 g/mol). To store peptides, before use in experiments: they were lyophilized and stored at −20°C.

### Preparation of fragments of the CB1a peptide

Three different fragments of CB1a were produced: F1 (front section): NH_2_-KWKVKKKIEKKWKV-COOH; F2 (middle section): NH_2_-WKVFKKIEKAG PKW-COOH; F3 (back section): NH_2_-KAGPKWKVFKKIEK-COOH. Fragments were synthesized, investigated and treated as previously described for CB1a (purity>95%).

### Production of monoclonal anti-CB1a antibodies

#### OVERVIEW

BALB/c mice were injected with the antigen: CB1a. B cells (B lymphocytes) were then isolated from mouse spleen and these were then fused with immortalized myeloma (B cell cancer) cells (using polyethylene glycol). The myeloma cells were selected beforehand to ensure they weren’t secreting antibody themselves and that they lack the hypoxanthine-guanine phosphoribosyltransferase gene. Fused cells were incubated on HAT (hypoxanthine-aminopterin-thymidine) medium. Aminopterin blocks the pathway for nucleotide synthesis but if a cell has a functioning HGPRT gene it can still produce nucleotides by the”salvage pathway”, using hypoxanthine and thymidine. If not, the cell will die and hence unfused myeloma cells die. Unfused B cells soon die because they have a short lifespan. Only hybrid cells survive and these are called hybridomas. These cells produce antibodies (a property of B cells) and are immortal (a property of myeloma cells). They produce only one type of antibody: monoclonal antibodies.

#### IN DETAIL

CB1a peptide was conjugated with Keyhole Limpet Hemocyanin (KLH) to produce CB1a-KLH. KLH is a large, multi-subunit, metalloprotein (protein with a metal ion cofactor) from a species of keyhole limpet and it is used here as a carrier protein for CB1a. Five BALB/c mice were intraperitoneally administered with CB1a-KLH two times (first with complete and second with incomplete Freund's adjuvant, which is an immunopotentiator) in order to provoke an immune response; that is to prompt the mice to produce antibodies against CB1a-KLH. Following each immunization, mice sera were tested using both anti-KLH IgG and anti-KLH IgM Enzyme-Linked Immunosorbent Assay (ELISA) kits. All mice showed a satisfactory immune response. The spleen was removed from one of these mice and macerated in ice for 5 minutes with 5 ml of red blood cell lysis solution (166 mM NH_4_Cl, 9 µM EDTA and 95 mM NaHCO_3_). The cell suspension (B lymphocytes) was then washed twice with RPMI medium and introduced to a BALB/c mouse myeloma cell line (NS-1), which was grown in RPMI 1640 medium supplemented with 20% fetal bovine serum. Cell fusion was initiated: mixing ratio was [5 lymphocyte: 1 myeloma] in a 1 ml polyethylene glycol/dimethylsulfoxide (1∶1; w/w) mixture for 1 minute which was then washed with RPMI medium for 5 minutes. Following fusion, the hybrid cells were re-suspended in RPMI medium (20% FCS, 40 µg/mL gentamicin, 1.25 µg/mL amphotericin B, 2 mM glutamine and 1 mM sodium pyruvate) before they were placed into wells (2.2×10^4^ cells per well; 72 wells) of a 96-well plate. The remaining 24 wells were filled with myeloma cells in HAT medium as controls. To eradicate non-fused cells in non-control wells: after 2 days growth, HAT medium was added to replace the original RPMI medium and further incubation was conducted for 10 days. The antibody-secreting hybridomas were screened by limiting dilution for 15 days. The desired hybridomas were further grown in HAT medium and six hybridoma cell lines derived from three parental clones were produced. Supernatants from hybridoma cell lines above were grown in HAT medium and six different anti-CB1a monoclonal antibodies were obtained (5C5H5, 5C5E8, 6G8D4, 6G8H3, 6D6H3, 6D6E7).

### Cells used

Normal lung cells (WI-38, MRC-5, HEL-299 cells lines), NSCLC (A549, NCI-H209, NCI-H460, NCI-H520 cell lines) and SCLC (NCI-H146 cell line) cells were purchased from the Bioresource Collection and Research Center (BCRC, Taiwan). They were cultured and grown at 37°C, in a humidified atmosphere with 5% CO_2_, in a medium containing RPMI 1640 (Gibco, CA, USA), 2 mM L-glutamine, 10 mM HEPES (4-(2-hydroxyethyl)-1-piperazineethanesulfonic acid), 1 mM sodium pyruvate, 4.5 g/l glucose, 1.5 g/l sodium bicarbonate and 10% fetal bovine serum (FBS, Gibco, CA, USA). Cell growth curves (number of cells versus time) were derived and when the cells were used in our experiments it is when they were at their log phases.

### Assaying the *in* *vitro* cytotoxicity of CB1a against cancer and normal cells

**Figure 1:**
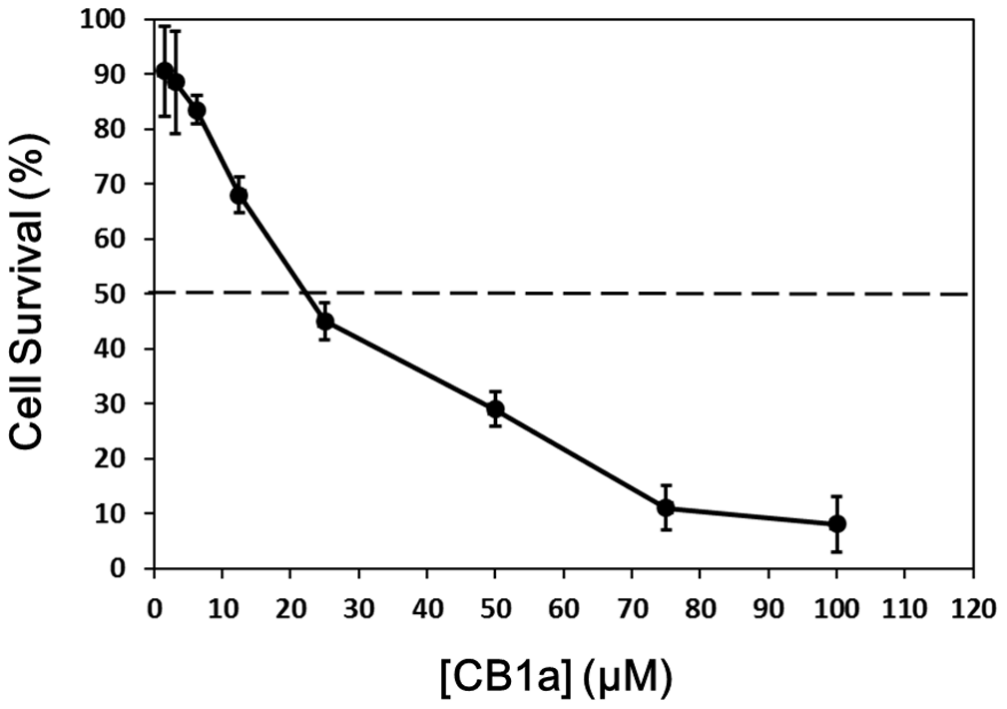
Plot to find the IC_50_ value for CB1a applied to NCI-H460 cancer cells. The initial cell concentration is 1×10^5^ cells. The greater the CB1a concentration, the lower the cell survival rate. IC_50_ (µM) is the CB1a concentration that produces a 50% cell survival rate. CB1a was applied once cells were in their log phase of growth (after ∼48 hours).

NCI-H460 cancer cell suspensions were made with 1×10^5^ cells/ml and this was put into a 96-well plate (90 µl/well). The suspension in each well was mixed with 10 µl of a culture medium containing different concentrations of CB1a peptide (1 µM to 200 µM); freshly prepared from 500 µM stock solutions. Plates were then incubated at 37°C in 5% CO_2_. After 2 days incubation, a 3-(4,5-Dimethylthiazol-2-yl)-2,5- diphenyl tetrazolium bromide (MTT)-based colorimetric assay was conducted. The mixture in each well had its absorbance at 560 nm (OD_560_) measured by a Bio-Rad model 450 microtiter plate reader. Then a calculation for each well with CB1a was made: (OD_560_ of cell sample incubated with CB1a) – (OD_560_ of control sample, incubated without CB1a). These experiments gave us a reading for the average NCI-H460 cell survival rate for different CB1a concentrations: the greater the CB1a concentration, the lower the cancer cell survival rate. IC_50_ is the concentration of CB1a that produces a 50% cell survival rate: for NCI-H460 cancer cells it is 22±3.5 µM (Figure 1).

This experiment with CB1a was then repeated but instead of using NCI-H460 cancer cells, other cell types were used in each further experiment: Normal lung cells (WI-38, MRC-5, HEL-299) and lung cancer cells: NSCLC (A549, NCI-H209, NCI-H520) and SCLC (NCI-H146). The results are shown in Table 1.

**Table 1:** IC_50_ (µM) is the concentration of CB1a that produces a 50% cell survival rate.

NLC			SCLCC	NSCLCC			
WI-38	MRC-5	HEL-299	NCI-H146	A549	NCI-H209	NCI-H460	NCI-H520
>300	84±3.5	158±6.8	4±0.6	29±4.3	5±1.4	25±3.5	5±0.8

IC_50_ is shown for normal lung cell (NLC) lines (WI-38, MRC-5, HEL-299), a small-cell lung cancer (SCLC) cell line (NCI-H146) and non-small-cell lung cancer (NSCLC) cell lines (A549, NCI-H209, NCI-H460, NCI-H520). IC_50_ is much lower for cancer cells than normal cells i.e. CB1a is much more toxic to cancer cells than to normal cells.

### Assaying if CB1a can inhibit tumor-like spheroid formation *in vitro*

We used non-adhesive, bacterial culture-grade polystyrene Petri dishes. Approximately 5×10^2 ^NCI-H460 cancer cells (3×10^4^ cells/ml) were deposited as a drop on the inner face of lids to 90 mm Petri dishes. These lids were then placed on their dishes, which were filled with 10 ml phosphate-buffered saline (PBS). A monolayer of NCI-H460 lung cancer cells was then de-attached by 0.25% Trypsin-EDTA. These dishes were then incubated at 37 C in a 5% CO_2_ incubator. This modified “hanging drop” method [48] causes the cancer cells to grow in a tumor-like aggregation; which is called a MCTS. After 3 days incubation, CB1a was applied in different concentrations (5, 15, 30, 45 and 60 µM) to different plates and a photo was then taken of each plate after 36 hours. This methodology investigated the post-treatment of CB1a, after tumor formation. The pre-treatment effect of CB1a, before tumor formation, was also studied. CB1a was applied in different concentrations (5, 15, 30, 45 and 60 µM) to different plates and left for 30 minutes before the 0.25% Trypsin-EDTA de-attachment step. After this step, the plates were incubated for 3 days and 36 hours before being photographed.

### Animal testing in nude strain mice

Animal experiments were conducted in a specific pathogen free environment as dictated by the Association for Assessment and Accreditation of Laboratory Animal Care International Guidelines. Four-week old male nude mice-NU/NU were used; sourced from BioLASCO Taiwan Co., Ltd., Taipei, Taiwan. We chose mice for our experiments that had a body weight of ∼30g. This value is ∼20% of the mean body weight of all our mice. It is prudent to experiment upon mice of a similar size, so that it isn’t a significant variable in our study. The mice were quarantined and allowed to adapt to an SPF room under 12 hour cycles of light and dark at 21±2°C and 60±20% humidity, while being fed *ad libitum* for 7 days. Afterwards, the animal test experiments were performed. Two groups of experiments were performed: pre-and- post-treatment of CB1a and post-treatment-only of CB1a; in relation to when the mouse was transplanted with human cancer cells. (a) pre-and-post-treatment of CB1a. Mice were randomly assigned to control and test groups, at 6 mice per group. Both groups were subcutaneously transplanted with human NCI-H460 cancer cells (1×10^6^ cells from suspended serum-free medium) at their abdominal flank. In the week prior to this, the control group was injected 3 times with saline, the test group was injected 3 times with CB1a. This was subcutaneously, in the dorsolateral neck area. The CB1a content in an injection depended on the weight of the mouse: 50 mg of CB1a was administered for every kg of the mouse (50 mg/kg); CB1a was administered in a liquid, dissolved in PBS (pH 6–7) at 5 mg/ml. Subsequently the control and test groups received their respective injections – saline or CB1a respectively −5 times a week for 5 consecutive weeks. Mouse body weights and tumor sizes (by caliper) were measured twice a week. Tumor volumes were calculated by: (major radius)×(minor radius)^2^/2(mm^3^). Forty-three days after the initial transplantation of tumor cells, the mice were sacrificed and their tumors extracted and weighed. (b) post-treatment-only of CB1a. 18 mice were equally divided into three groups. They were all were subcutaneously transplanted with human NCI-H460 cancer cells (2×10^6^ cells from suspended serum-free medium) at their abdominal flank. The mice were left until their tumor sizes reached around 30∼70 mm^3^. One group was injected with docetaxel, which is a drug in present clinical use against lung cancers. Another group was injected with CB1a and the last with saline. All injections were intravenous and each group received 5 injections per week for 4 consecutive weeks. In a CB1a solution, 50 mg of CB1a was introduced for every kg of the mouse (50 mg/kg); CB1a was dissolved in PBS at 5 mg/ml. In a docetaxel solution, 10 mg of CB1a was introduced for every kg of the mouse (10 mg/kg); docetaxel was dissolved in PBS at 2 mg/ml. The solutions were administered such that the test mice were given the same molar concentration of either CB1a or docetaxel in each injection (0.012 mmol/kg).

### Using ELISA to elucidate the lifespan of CB1a in rat blood

ELISA is a test that uses antibodies and a color change to detect the presence of a substance in a liquid sample [49]. There are different variations to this technique but we used the following to detect CB1a in a sample: the sample is immobilized on a solid support via adsorption to the surface. The detection/primary antibody is added and it forms a complex with its antigen (CB1a), if it present in the sample. The secondary antibody, bound by the horseradish peroxidase enzyme (HRP), is then added and it forms a complex with the primary antibody. A substrate for HRP is then added – tetramethylbenzidine - and as HRP oxidases it a color change occurs. H_2_SO_4_ is added to stop the reaction. The amount of antigen (CB1a) is coded in the color change and it is quantified by assaying the absorption of light at 450 nm (OD_450 nm_). Between each step, the plate is washed with a detergent solution to remove any proteins or antibodies that are a specifically bound. Blocking buffer is added before the antibody steps to block non-specific binding. It binds to any part of the plate not occupied by antigen (CB1a), so then the primary antibodies can only bind to the plate via a binding with the antigen. There is no place free for them to bind directly in a non-specific manner.

As aforementioned we produced a number of different antibodies against CB1a: 5C5H5, 5C5E8, 6G8D4, 6D6H3, 6D6H3, 6D6E7, 6G8H3. We wanted to find which of these would bind only complete CB1a and none of its separated, composite fragments: F1, F2, F3. So, we tested the different antibodies against complete CB1a (1 µg/ml) and three different CB1a fragments: F1, F2, F3 (each at 100 µg/ml) in ELISA experiments.

CB1a peptide, diluted with phosphate buffered saline (PBS; 2.5 µg/ml), was added to an ELISA plate (100 µl/well; 1 µg/ml) and left overnight at 4°C. The same procedure was done for each of the CB1a fragments: F1, F2, F3 (100 µl/well; 100 µg/ml). Note that the concentrations of fragments used were 100 fold higher than that used for CB1a. After washing with washing buffer 5 times (phosphate buffered saline tween, PBST): 200 µl blocking buffer (1% bovine serum albumin, BSA, in PBS) was added into the wells and they were left for 1 hour at room temperature. They were then washed with PBST buffer again 5 times. Then different dilution rates of the primary antibody were added (100 µl/well; dilution rates: 1∶1000, 1∶2000, 1∶5000, 1∶10,000; diluent buffer was 1% BSA and 0.05% Tween 20 in PBS) and were then left for 1 hour at room temperature. The best dilution rate was 1∶5000 (data not shown). Then, after washing with PBST buffer 5 times, the secondary antibody (goat anti-mouse IgG) conjugated with enzyme horseradish peroxidase (HRP; HRP-secondary antibody), in a diluent buffer (100 µl/well), was added and left for 1 hour at room temperature. The best dilution rate for the addition of secondary antibody was 1∶2000. After washing with PBST buffer a further 5 times, 100 µl tetramethylbenzidine was added for 20 minutes (color developing). Finally, 50 µl of 2M H_2_SO_4_ was added to stop the reactions. Results at OD_450 nm_ were recorded using an automated ELISA reader.

For all the antibodies tested – their affinity to the complete CB1a was much greater than their affinity to the fragments. This differential was highest with the 5C5H5 antibody and it is so high that we can pretty much assume that any binding to this antibody is complete CB1a and not its broken fragments. We used this antibody for all subsequent ELISA experiments.

**Figure 2:**
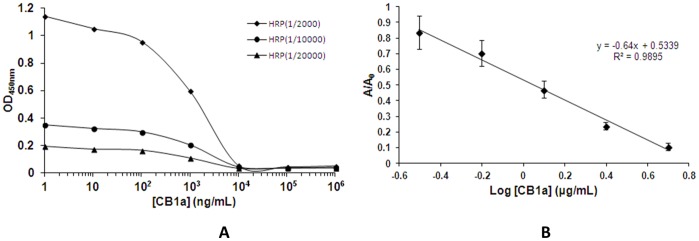
The establishment of a standard curve, which can be used to look up the CB1a concentration of a test sample from its measured adsorption. If an ELISA experiment is repeated with different solutions of known CB1a concentration; a calibration curve can be made, which shows the OD_450nm_ (A/A_0_) values produced by different, given CB1a concentrations. Using this standard curve, an unknown concentration of CB1a in a test sample can then be found by finding its OD_450nm_ (A/A_0_) value in an ELISA experiment. (*A*) The absorbance at 450 nm (OD_450nm_) for different dilution rates (1∶2000; 1/10,000; 1/20,000) of secondary antibody (conjugated with horseradish peroxidase, HRP) and known concentrations of CB1a (ng/ml). (*B*) Log [CB1a] versus ELISA absorption (A/A_0_); with 1/2000 dilution rate of secondary antibody used. The plotted line is y = −0.64x+0.5339. This is our calibration curve.

If an ELISA experiment is repeated with different solutions of known CB1a concentration; a calibration curve can be made, which shows the OD_450nm_ values produced by different, given CB1a concentrations. Using this standard curve, an unknown concentration of CB1a in a test sample can then be found by finding its OD_450nm_ (A/A_0_) value in an ELISA experiment. Figure 2A shows different standard curves produced when using different dilution rates of secondary antibody (1∶2000; 1/10,000; 1/20,000). For our definitive standard curve, we used the one with a dilution rate of 1∶2000 and utilized this dilution rate when assaying test blood samples. We used a primary antibody dilution rate of 1∶5000. The standard curve is a straight line when we use the logarithm of CB1a concentration; Figure 2B shows log[CB1a] versus ELISA absorption at 450 nm (A/A_0_).

Six four-week-old male Sprague-Dawley rats (230–260 g; sourced from BioLASCO, Yilan, Taiwan) were housed (3 per cage) in a specific pathogen free animal room. The rats were intravenously injected with CB1a peptide (50 mg/kg) and blood samples were then collected from the tail vein of the rats at the following time points: 5, 20, 25, 38.5, 50, 60 and 240 minutes after the CB1a injection (6 samples taken at each of these time points). The blood samples were transferred to heparinized microcentrifuge tubes and centrifuged at 4,000 g for 5 minutes; then frozen for storage at −80°C.

The blood samples were analyzed via a competitive ELISA technique [49], rather than the more direct ELISA technique described earlier. Blood samples were diluted 100 fold and then mixed with a known concentration of CB1a antibody (2mg/ml in PBST buffer, including 1% BSA). These samples were then incubated at 37°C for 2 hours, to give a chance for the antibodies to bind the CB1a peptides. The samples were then added to a CB1a coated plate [*] (100 µl/well) and incubated for 1 hour at room temperature. The primary antibodies not already sequestered by CB1a in the sample, from the previous step, bind to this plate. Afterwards, the sample is washed off the plate (washed 5 times with PBST buffer). The primary antibodies that are in a complex with the free CB1a in the sample, and not with the CB1a on the plate, or unbound antibodies are washed away. Then secondary antibodies, conjugated with enzyme horseradish peroxidase, are added and the process proceeds as described previously. The CB1a concentration recorded on the plate is added to the known CB1a concentration that is washed off, bound to the CB1a antibodies, to get the total CB1a concentration.

[*] This CB1a coated plate was prepared as follows: CB1a was added to an ELISA plate and the plate was then washed 5 times with PBST buffer to remove unbound CB1a. Any part of the plate not bound by CB1a was then blocked by the binding of BSA in an introduced blocking buffer (1% BSA in PBS, 100 µl/well), left for 1 hour at room temperature before being washed off by 5 washes with PBST buffer.

## Results

### CB1a can kill cancer cells without killing normal cells *in* *vitro*

In separate experiments, *in* *vitro*, CB1a was applied to normal lung cells: with a number of different cell lines: WI-38, MRC-5, HEL-299. Similarly, CB1a was applied to SCLC cells (NCI-H146) and to NSCLC cells (A549, NCI-H209, NCI-H460, NCI-H520). For all these cells: the greater the CB1a concentration, the lower the cell survival rate. IC_50_ is the concentration of CB1a that produces a 50% cell survival rate. The CB1a concentration required to kill the cell was much higher for normal cells than for cancerous cells. That is to say the IC_50_ was much higher for normal cells than cancer cells (Table 1). So, CB1a has a much greater lethality upon cancer cells than normal cells. It can kill cancer cells at concentrations that do not kill normal cells; in this sense it can have a specific kill action against cancer cells.

**Figure 3:**
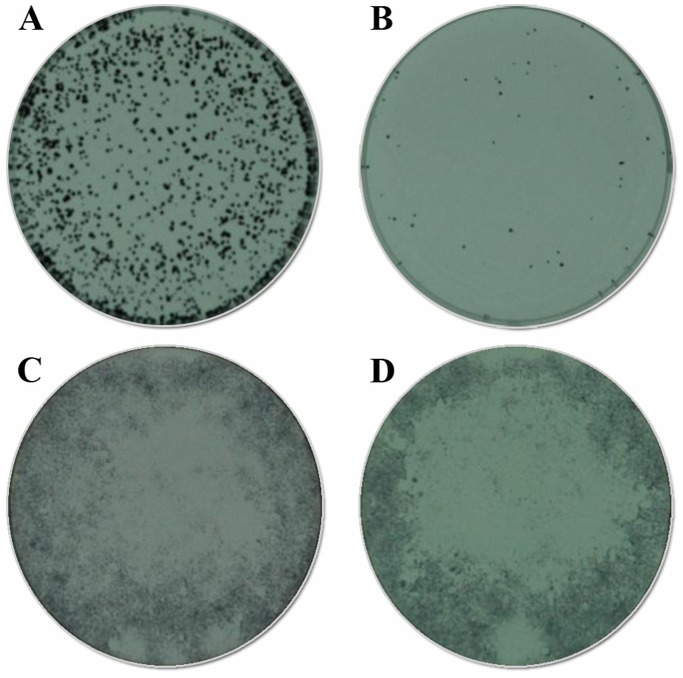
*In vitro*, CB1a kills cancer cells at a concentration that does not kill normal cells. Photos A and B show without and with CB1a (50 µM), on NCI-H460 cancer cells, respectively. Photos C and D show without and with CB1a (50 µM), on MRC-5 normal cells, respectively. Initial cell concentrations on plates were 2×10^3^ cells for all; photos were taken after 5 days incubation; where CB1a was applied it was done so at the end of day 4. Comparing A and B: one can note without CB1a there is significant cellular growth, as to be expected from a cancer cell line; with CB1a there is not, presumably because of a CB1a kill action against cancer cells. C and D have a comparable number of cells, presumably because CB1a does not kill normal cells at this concentration.

NCI-H460 cancer cells were grown on two cell plates (2×10^3^ cells at start in each) – after 4 days, CB1a (50 µM) was added to one and not the other. After 24 hours these 2 plates were photographed: the one without CB1a (Figure 3A) showed significant cell growth as one would expect of a cancerous cell line; the one with CB1a present had significantly less cells, presumably because of the kill action of CB1a against cancer cells (Figure 3B). This experiment was repeated but with normal cells. MRC-5 normal cells were grown on two cell plates (2×10^3^ cells at start in each) – after 4 days, CB1a (50 µM) was added to one and not the other. After 24 hours these 2 plates were photographed: the one without CB1a (Figure 3C) had a comparable number of cells to the one with CB1a present (Figure 3D). This indicates that CB1a application, at this concentration, does not kill these normal cells. Overall, this cell plate study shows that CB1a kills cancer cells at a concentration (50 µM) that does not kill normal cells.

### CB1a can disrupt tumor-like spheroids in test tubes

**Figure 4:**
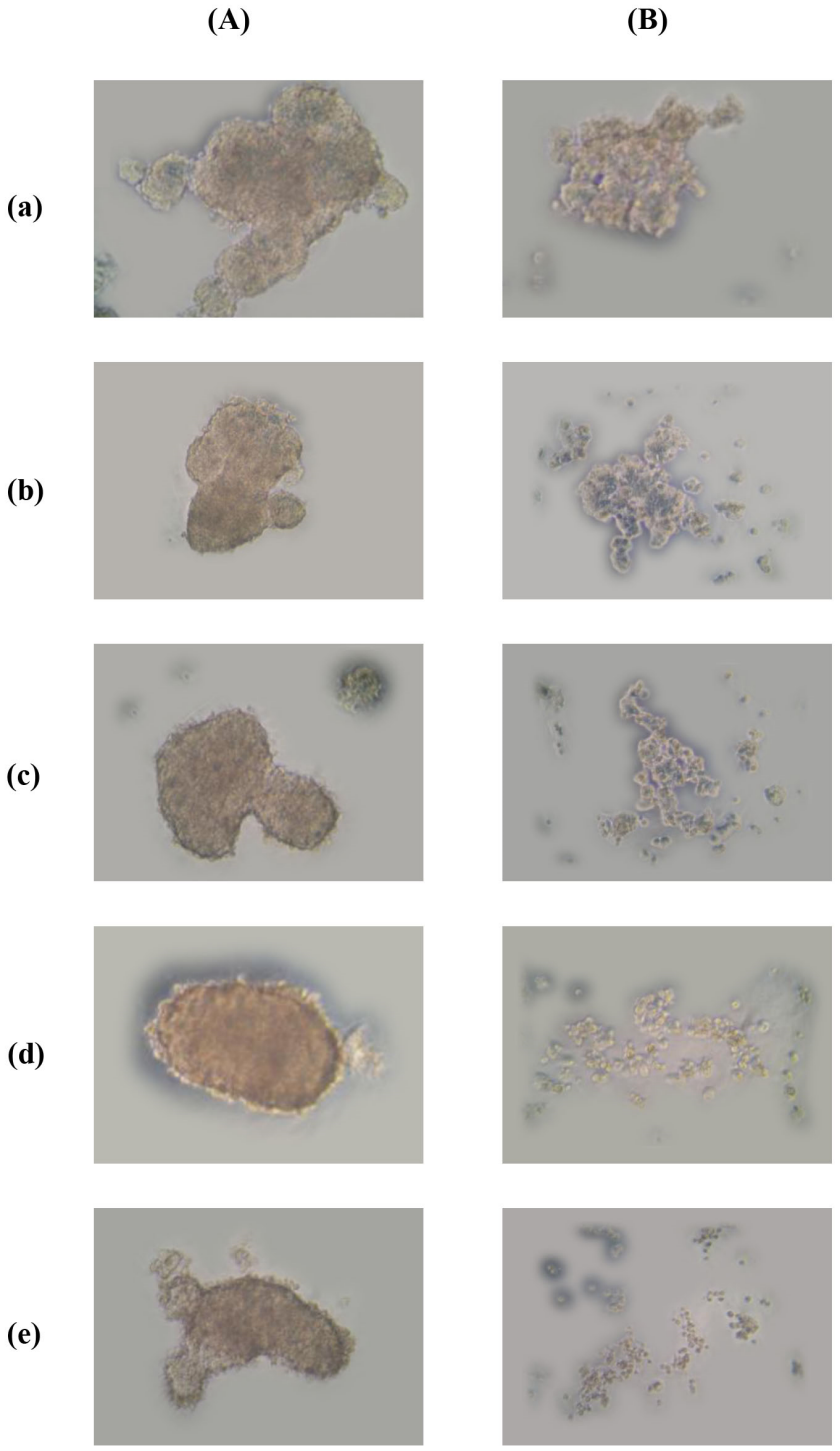
*In vitro,* CB1a can disrupt lung cancer cells from adhering together to form tumor-like spheroids. 5×10^2^ NCI-H460 lung cancer cells were introduced to each cell plate (hanging drop method, refer *Methods*). In the left column (*A*), plates were “pre-treated” with CB1a before the introduction of cells. In the right column (*B*), plates were “post-treated” with CB1a after the introduction of cells. The concentration of CB1a applied is increased as we go vertically down the columns: 5, 15, 30, 45 and 60 µM for panels a, b, c, d and e respectively. With post-treatment, spheroids are still observed. With pre-treatment: the greater the CB1a concentration used, the smaller the spheroid; until - with higher CB1a concentrations - no spheroids form. High CB1a concentrations can block cancer cell adhesions.

Figure 4 shows photos of NCI-H460 lung cancer cells on cell plates. In the left column they have been post-treated with CB1a (for 36 hours) after the introduction of a fixed number of cancer cells (5×10^2^). In the right column, the plates have been pre-treated with CB1a before the introduction of the same number of cancer cells, and then photographed after 36 hours. The concentration of CB1a used is increased as we go vertically down the columns: 5, 15, 30, 45 and 60 µM for panels a, b, c, d and e respectively. In the case of post-treatment, tumor spheroids can still be observed. In the case of pre-treatment: the greater the CB1a concentration used, the smaller the spheroid; until - with higher CB1a concentrations - no spheroids form. High CB1a levels can inhibit the ability of cancer cells to adhere to one another to form tumor-like spheroids; cancer cells are still present but in lower numbers and are less associated.

### CB1a can inhibit the growth of lung tumors in an *in* *vivo* mouse model

**Figure 5:**
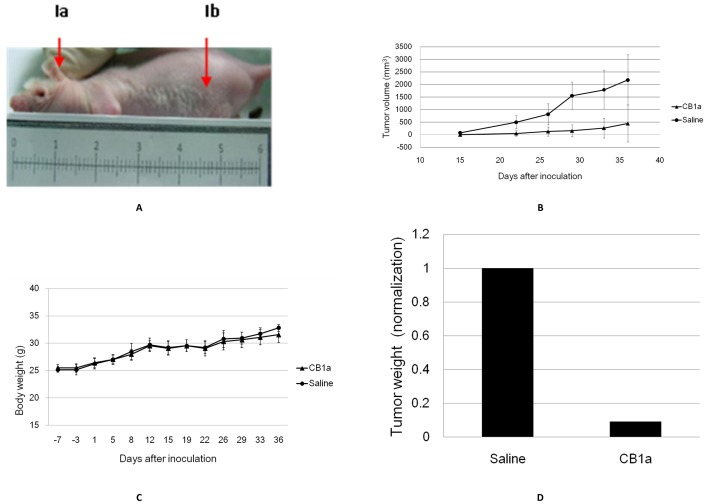
CB1a can inhibit the growth of lung tumors in an *in* *vivo* mouse model. Six mice were subcutaneously pre-treated with CB1a (50 mg/kg) for one week prior to them being subcutaneously xenografted with human tumorigenic NCI-H460 lung cancer cells (0.2 ml; 1×10^6^ cells/ml). A control was studied in which saline was used instead of CB1a (6 mice in this control group). (*A*) The CB1a/saline injection point was in the dorsolateral neck area (arrow labelled *Ia*) and the cancer cells were transplanted to the abdominal flank area of the mouse (arrow labelled *Ib*). The ruler shows that these points are ∼4 cm apart, which is long relative to the length of the mouse (∼6 cm). (*B*) Mean body weight (g) vs. time (days); the mean body weight of CB1a treated mice (triangles) was comparable to that of saline treated mice (circles), which indicates that CB1a is not overly toxic to normal cells. (*C*) Mean tumor volume (mm^3^) vs. time (days) for CB1a (triangles) or saline (circles) treated mice. The tumor is much smaller in CB1a treated mice; after 36 days the mean tumor volume in CB1a treated mice is just 19.3% of that in saline treated mice. (*D*) The mice were sacrificed on day 36 in order for their tumors to be weighed. Mean tumor weight in saline treated mice normalized as 1.0; the mean tumor weight of CB1a treated mice is just 9% of this value.

Six mice were pre-treated with CB1a (50 mg/kg) 3 times in one week prior to them being subcutaneously xenografted with human tumorigenic NCI-H460 lung cancer cells (0.2 ml; 1×10^6^ cells/ml). A control was studied in which saline was used instead of CB1a (6 mice in this control group). The dose of CB1a/saline was continued after the xenograft: 5 times per week for 5 weeks. The CB1a/saline injection point was in the dorsolateral neck area and the xenograft cancer cells were transplanted into the abdominal flank area of the mouse (Figure 5A). The distance between these two points is far (∼4 cm) as compared to the length of the mouse (∼6 cm). This distance tested whether CB1a could effectively travel in the blood stream without being destroyed. None of the mice died during this experiment. The mean body weight of CB1a treated mice was comparable to that of saline treated mice (Figure 5B), which indicates that CB1a is not overly toxic to normal cells. 36 days after the human lung cancer cells were xenografted on to the mouse – the mean volume of the tumor in the CB1a treated mice was just 19.3% of the tumor in the saline treated mice (Figure 5C); the mean weight of the tumor in CB1a treated mice was just 9% of the tumor in saline treated mice (Figure 5D). In other words, the tumor was ∼80% smaller and ∼90% lighter in CB1a treated mice.

### CB1a is more toxic to cancer cells, and less toxic to normal cells, than docetaxel in an *in* *vivo* mouse model

**Figure 6:**
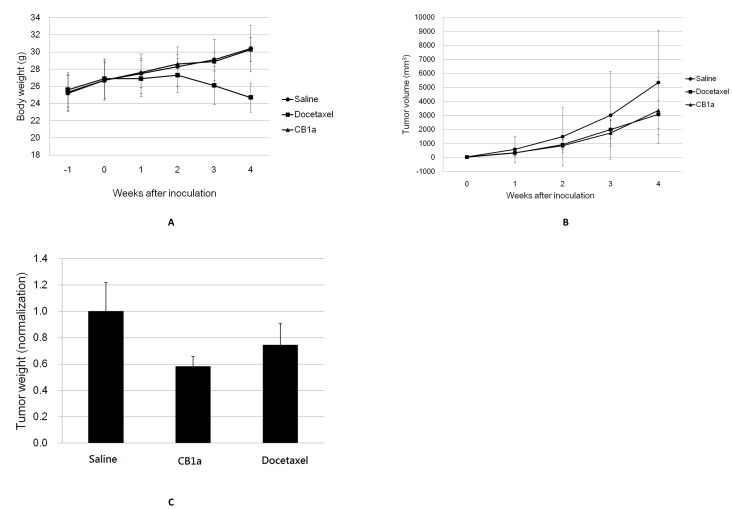
CB1a is more toxic to cancer cells, and less toxic to normal cells, than docetaxel in an *in* *vivo* mouse model. Mice were subcutaneously injected, at their abdominal flank, with human tumorigenic NCI-H460 cancer cells (0.2 ml; 2×10^6^ cells/ml) [at “week 1”]. After a week, [at “week 0”], these mice had tumors with a volume of 30–70 mm^3^. Six of these cancerous mice were then intravenously injected with a course of docetaxel, six more were intravenously injected with a course of CB1a instead (at the same molar concentration; 0.012 mmol/kg) and a further six of these mice were intravenously injected only with a course of saline solution. (*A*) The body weight of these mice was then monitored and the mean weight (g) of each grouping was plotted against time (week). CB1a treated mice (triangles) gained weight at the same trajectory as saline treated mice (circles), in contrast to the weight loss observed with docetaxel treated mice (after week 2; squares). CB1a is less toxic to normal cells and physiology than docetaxel. (*B*) Mean tumor volume (mm^3^) vs. time (week) for CB1a (triangles), docetaxel (squares) and saline (circles) treated mice. The tumor volume increased at a similar shallow trajectory for both CB1a and docetaxel treated mice, much less than in saline treated mice. At week 4, the mean tumor volume was 5,400 mm^3^ in saline treated mice, 3,200 mm^3^ in docetaxel treated mice and 3,100 mm^3^ in CB1a treated mice. (*C*) The mice were sacrificed at week 4 in order for their tumors to be weighed. Mean tumor weight in saline treated mice normalized as 1.0; the mean tumor weight of docetaxel and CB1a treated mice is 73% and 59% of this value respectively. CB1a is more toxic to cancer cells than docetaxel.

Docetaxel is a drug that is licensed for, and can be effective against, human lung cancers; however, it has a bad side effect profile. Presumably, because its toxicity is not specific enough to cancer cells and it adversely affects normal cells also. Despite this it is in present clinical use because of a lack of alternatives. We compare the action of CB1a to that of docetaxel. Mice were subcutaneously injected, at their abdominal flank, with human tumorigenic NCI-H460 cancer cells (0.2 ml; 2×10^6^ cells/ml) [at “week 1]. After a week, [at “week 0], these mice had tumors with a volume of 30–70 mm^3^. Six of these cancerous mice were then intravenously injected with docetaxel, six more were intravenously injected with CB1a instead (at the same molar concentration; 0.012 mmol/kg) and a further six of these mice were intravenously injected only with saline solution. The body weight of these mice was then monitored for the next 5 weeks; the mean weight of each grouping was then plotted (Figure 6A). Docetaxel treated mice reached a maximum body weight at week 2 (27 g) before losing weight: 25 g by week 4 (Figure 6A) and 21 g by week 5 (data not shown). CB1a treated mice were 25.5 g at the start and gained weight throughout: to be 30.5 g at week 4 (Figure 6A) and 32 g at week 5 (data not shown). In fact, CB1a treated mice gained weight at the same trajectory as saline treated mice, in contrast to the weight loss observed with docetaxel treated mice (Figure 6A). This indicates that CB1a is less toxic than docetaxel to normal cells. By contrast, we find that CB1a is *more* toxic than docetaxel to cancer cells. At week 4– the end of the experiment - the mean tumor volume was 5,400 mm^3^ in saline treated mice, 3,200 mm^3^ in docetaxel treated mice and 3,100 mm^3^ in CB1a treated mice (Figure 6B). At week 4 the mice were sacrificed so that their tumors may be weighed. The mean tumor weight in docetaxel treated mice was 73% of the mean tumor weight in saline treated mice; the mean tumor weight in CB1a treated mice was 59% of the mean tumor weight in saline treated mice (Figure 6C). So, to conclude, CB1a is more toxic to cancer cells, but less toxic to normal cells, than docetaxel.

### Pharmacokinetics of CB1a in rat blood

**Figure 7:**
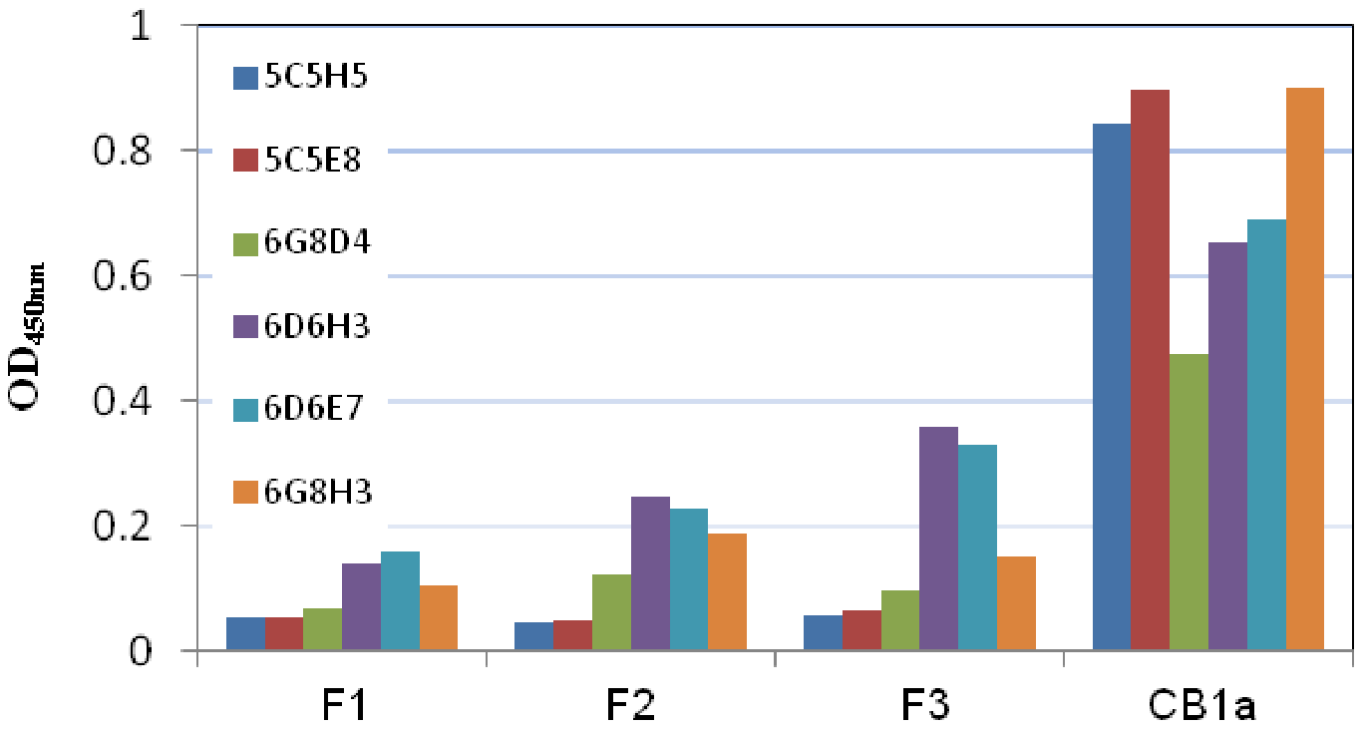
Experiment to find an antibody that binds only complete CB1a and none of its separated, composite fragments: F1, F2, F3. We tested different antibodies (5C5H5, 5C5E8, 6G8D4, 6D6H3, 6D6H3, 6D6E7, 6G8H3; dilution rate of 1∶2000) against complete CB1a (1 µg/ml) and three different CB1a fragments: F1, F2, F3 (each at 100 µg/ml; refer Methods). For all the antibodies tested – their affinity to the complete CB1a was much greater than their affinity to the fragments. This differential was highest with the 5C5H5 antibody and it is so high that we can pretty much assume that any binding to this antibody is complete CB1a and not its broken fragments.

**Figure 8:**
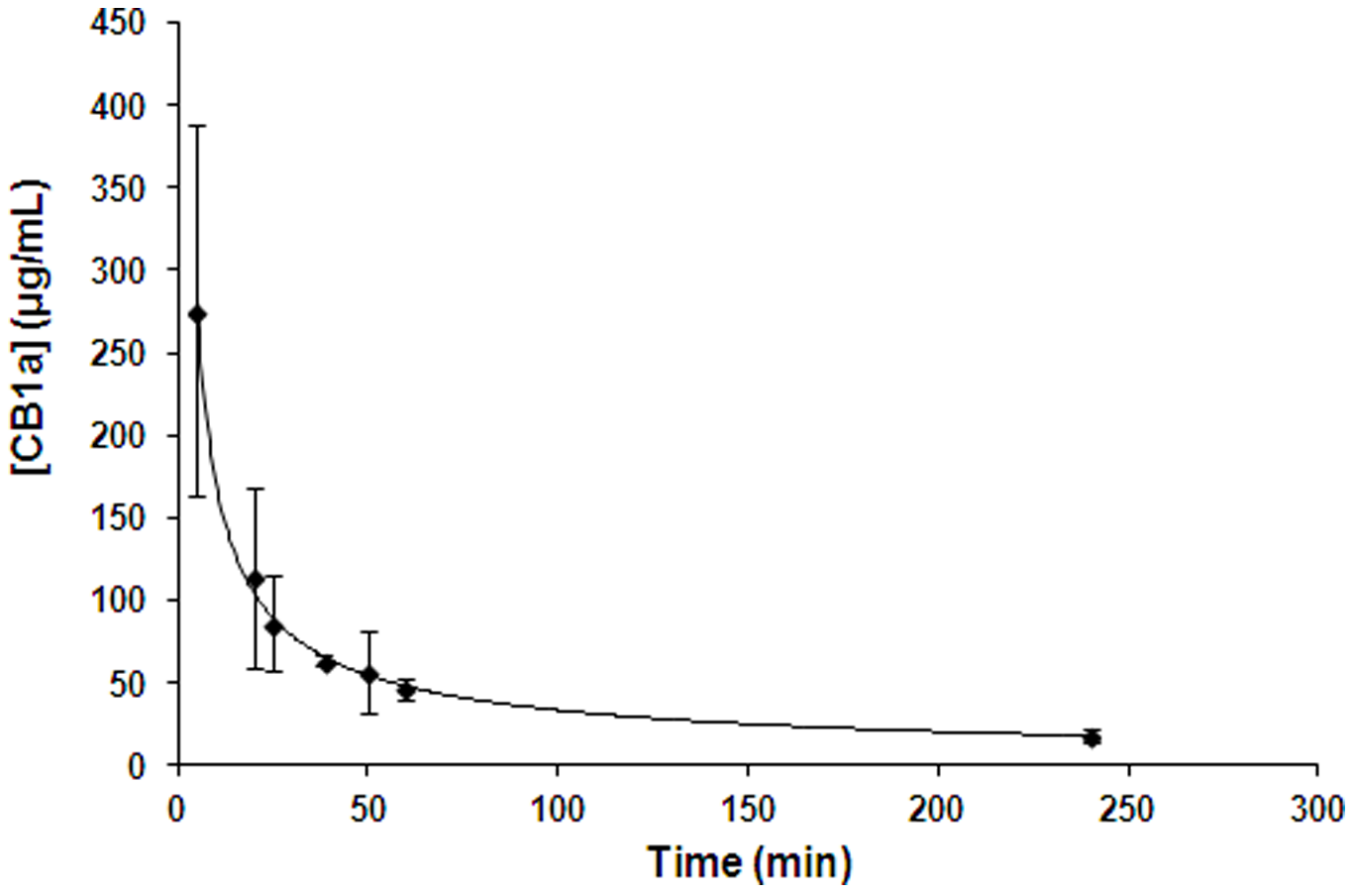
Pharmacokinetics of CB1a in rat blood. CB1a was injected into rats and their blood was drawn at subsequent time points (6 times at each point) and the 5C5H5 antibody was used in an ELISA experiment to assay how much complete CB1a remained in the blood stream. The standard curve in Figure 2B was used. We found that the half-life of CB1a in rat blood is 16.4 minutes.

We wanted to find out how long CB1a exists in the rat bloodstream. To do this we sought an antibody that has a high binding affinity for the complete CB1a peptide but not to its composite fragments, which are released when it is broken up by proteases. We tested a number of different antibodies against complete CB1a (1 µg/ml) and three different CB1a fragments: F1, F2, F3 (each at 100 µg/ml; refer Methods). For all the antibodies tested – their affinity to the complete CB1a was much greater than their affinity to the fragments (Figure 7). This differential was highest with the 5C5H5 antibody and it is so high that we can assume that any binding to this antibody is complete CB1a and not its broken fragments. We used this antibody for the next experimental step. CB1a was injected into rats and their blood was drawn at subsequent time points (6 times at each point) and the 5C5H5 antibody was used to assay how much complete CB1a remained in the blood stream (Figure 8). We found that the half-life of CB1a in rat blood is 16.4 minutes. A CB1a concentration that can kill cancer cells (≥25 µM or ≥105 µg/ml) endures for around 25 minutes which is long enough: the time required for CB1a to kill a cancer cell is between 15 and 20 minutes (data not shown). The half-life of CB1a is likely to be even longer in humans.

## Discussion

### CB1a toxicity can be selective to cancer cells

*In vitro*, CB1a can kill lung cancer cells at concentrations that do not kill normal lung cells. Or indeed other normal cells tested: kidney HFL1 cells, 3T3/3T6 tissue cells etc. (data not shown). IC_50_ is the chemical concentration that produces 50% cell survival. The selectivity index of a lung cancer drug is a measure of how specific its killing action is to cancer cells. It is the ratio of its IC_50_ for a normal lung cell to its IC_50_ for a cancerous lung cell. The value for CB1a, calculated from the data in Table 1, is between 10 and 60. So, it is much more selective than drugs presently on the market, which generally have an SI of less than one. For example, docetaxel or doxorubicin. The poor selectivity of these drugs produces severe side effects. We show that CB1a is more toxic to cancer cells, and less toxic to normal cells, than docetaxel in an *in* *vivo* mouse model. Tumors were smaller in CB1a treated mice and these mice gained weight at the same rate as a saline control group. Whereas docetaxel treated mice lost weight, indicating side effects and physiological damage. Particularly good results could be seen with CB1a if its treatment course was started before the introduction of cancerous cells. The resulting tumors were ∼80% smaller and ∼90% lighter on average in CB1a treated mice than in a saline treated control group. So, CB1a may particularly excel as a treatment to stop the return of a tumor after one has been removed by surgery; or indeed after prior chemotherapy, radiotherapy or some combination of these treatments.

### CB1a can survive in the bloodstream long enough to exert its therapeutic action

In our mouse study, the human lung cancer cells were injected subcutaneously into the mouse’s abdominal flank and the drug, CB1a peptide, was infused subcutaneously into the dorsolateral neck of the mouse (Figure 5A). The distance between these two injection sites is far as compared to the length of the mouse. The positive action of CB1a shows that it can travel in the blood and last long enough to exert an effect, before it is digested by proteases in the blood serum. One reason for this longevity may be CB1a’s design of three repeated, amphipathic sequences. CB1a has a half-life in rat blood of about 16.4 minutes. A cancer killing concentration of CB1a can persist in rat blood for long enough to kill cancer cells. The half-life of this peptide in humans is likely to be longer.

### CB1a may potentiate the action of other cancer therapeutics and combat multicellular resistance (MCR)

*In vitro*, cancer cells can aggregate into spheroids and exhibit a phenomenon known as MCR. Tumors in patients can also present MCR [50]. So, it is important to test anti-cancer drugs against tumor-like spheroids rather than monolayer cell cultures. Spheroid structure hides and protects inner cancer cells from the action of applied therapeutics [51]. Furthermore, inner cells are in a hypoxic and necrotic center and can be non-proliferatory, which makes them immune to drugs that target cycling cells. This contact-dependent resistance can be eliminated if cell contacts are disrupted. We have shown CB1a’s ability to disrupt MCTS growth *in* *vitro*. In these assays, pre-dosing with CB1a could disrupt cancer cell association and spheroid formation. Although these *in* *vitro* experiments cannot wholly mimic real tumor growth, we go on to show that CB1a can prevent tumor growth *in* *vivo*. CB1a’s ability to prevent tumor growth - by corrupting the adhesion among cancer cells – may combat MCR by opening up and restoring killing pathways for other drugs rendered impotent by MCR effects.

### Mechanism of CB1a action

**Figure 9:**
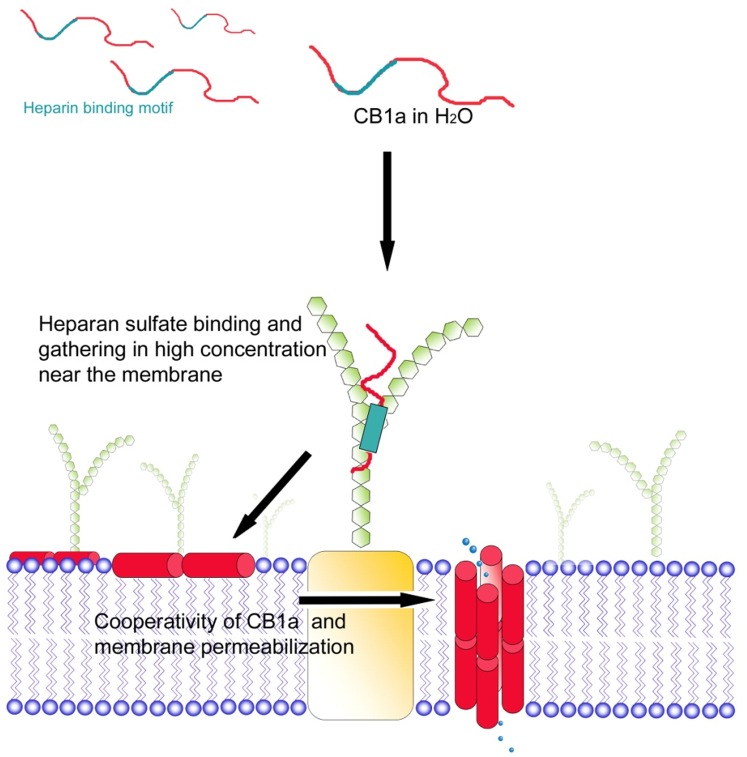
How does CB1a kill a cancer cell? A postulated mechanism. CB1a is unstructured in an aqueous solution, but adopts a helical conformation in a membrane-like environment. CB1a has a heparin binding motif and this binds a heparan sulfate proteoglycan sticking out of the cell surface. In a second step, the amphipathic (one side hydrophilic, other side hydrophobic) sections of CB1a interact with the hydrophilic polar heads, and then the hydrophobic tails, of the membrane lipid bilayer. CB1a is incorporated into the membrane, as a transmembrane pore. This foreign, sabotaging pore then results in programmed cell death (apoptosis).

CB1a is unstructured in an aqueous solution, but adopts a helical conformation in a membrane-like environment [52]. CB1a has a heparin binding motif (EKKWKV) aimed to bind with heparan sulfate proteoglycans (HSPGs) on cell surface (Figure 9). HSPGs are an important component of the cancer tumor extra-cellular matrix [53]. In a second step, the amphipathic (one side hydrophilic, other side hydrophobic) sections of CB1a interact with the hydrophilic polar heads, and then the hydrophobic tails, of the membrane lipid bilayer. CB1a may be incorporated into the membrane; possibly as a transmembrane entity. This may then cause a pore formation in the membrane, with the proline residue in the AGP hinge bridge motif possibly involved in pore gating [54]. The ensuing damage/problems may set in motion programmed cell death (apoptosis).

In a previous report we showed that the mechanism for cell death under CB1a toxicity was verified as being largely due to cell surface damage. CB1a damages the surface of cancer cells but not normal cells [45]; possibly because it binds to the surface of cancer cells better than it does to normal cells. This may be because cancer cells have more HSPGs at their surface [53]; and/or because CB1a is very positive/cationic (+12) and cancer cells could have a more negatively charged membrane, possibly because of a higher phosphatidylserine composition [55]. The damage of cancer cell surface (including HSPGs) may lower the ability of cell adhesion [56] leading to reduce the formation of tumor (Figure 4).

CB1a binds to cells via their lipid bilayer and not at specific membrane protein receptors. This is distinct from protein anti-cancer drugs like gefitinib that target protein receptors (e.g. epidermal growth factor receptor). Arguably, the potential for drug resistance is much higher with these specific targets than with CB1a, which targets the more ubiquitous lipid bilayer. CB1a has a distinct killing pathway, which makes it a valuable prospect.

### Peptides as therapeutics

Peptides have the potential to be a new generation of therapeutics. Indeed, some have already been successfully commercialized as drugs: (a) enfuvirtide (36 amino acids) is an HIV fusion inhibitor [57]; (b) bivalirudin (20 amino acids) is a thrombin inhibitor/anti-coagulate [58]. CB1a is a peptide and we show that it has significant potential as a therapeutic. It merits further investigation. In conclusion, *in* *vitro* and *in* *vivo* models indicate that CB1a may be an effective treatment for human lung cancer.
